# Epigenetic aging in older people living with HIV in Eswatini: a pilot study of HIV and lifestyle factors and epigenetic aging

**DOI:** 10.21203/rs.3.rs-3389208/v1

**Published:** 2023-10-05

**Authors:** Christian K. Dye, Haotian Wu, Gabriella L. Jackson, Altaye Kidane, Rejoice Nkambule, Nomthandazo G Lukhele, Bongiwe Prudence Malinga, Rhinos Chekenyere, Wafaa M. El-Sadr, Andrea A. Baccarelli, Tiffany G. Harris

**Affiliations:** Columbia University Mailman School of Public Health; Columbia University Mailman School of Public Health; Columbia University Mailman School of Public Health; ICAP at Columbia University; Eswatini Ministry of Health; Eswatini Ministry of Health; Eswatini Ministry of Health; Eswatini Ministry of Health; ICAP at Columbia University; Columbia University Mailman School of Public Health; ICAP at Columbia University

**Keywords:** Epigenetics, epigenetic aging, biological aging, HIV, epigenetic age acceleration, DunedinPACE, epigenetic clock, older people living with HIV, Africa, Eswatini

## Abstract

**Background:**

People living with HIV (PLHIV) on effective antiretroviral therapy (ART) are living near-normal lives. Although they are less susceptible to AIDS-related complications, they remain highly vulnerable to non-communicable diseases (NCD). In this exploratory study of older PLHIV (OPLHIV) in Eswatini, we investigated whether biological aging (*i.e.*, the difference between epigenetic age and chronological age, termed ‘epigenetic age acceleration [EAA]’) was associated with HIV-related parameters, and whether lifestyle factors modified these relationships. We calculated EAA focusing on the second-generation epigenetic clocks, PhenoAge and GrimAge, and a pace of aging biomarker (DunedinPACE) among 44 OPLHIV in Eswatini.

**Results:**

Among participants, the PhenoAge clock showed older epigenetic age (68 years old [63, 77]) but a younger GrimAge epigenetic age (median=56 years old [interquartile range=50, 61]) compared to the chronological age (59 years old [54, 66]). Participants diagnosed with HIV at an older age showed slower DunedinPACE (β-coefficient [95% Confidence Interval]; −0.02 [−0.04, −0.01], **p**=0.002) and longer duration since HIV diagnosis was associated with faster DunedinPACE (0.02 [0.01, 0.04], **p**=0.002). The average daily dietary intake of fruits and vegetables was associated with faster DunedinPACE (0.12 [0.03, 0.22], **p**=0.01) and modified the relationship between HIV status variables (number of years living with HIV since diagnosis, age at HIV diagnosis, CD4^+^ T cell counts) and PhenoAge EAA, and DunedinPACE.

**Conclusions:**

Biological age is accelerated in OPLHIV in Eswatini, with those living with HIV for a longer duration at risk for faster biological aging. Lifestyle factors, especially healthier diets, may attenuate biological aging in OPLHIV. To our knowledge, this is the first study to assess biological aging in Eswatini and one of the few in sub-Saharan Africa.

## 1. Introduction

The advent of highly effective antiretroviral therapy (ART) has shifted the landscape of HIV from a deadly disease to a chronic condition, often associated with multi-morbidities (1). Access to ART and viral load suppression is associated with decrease in morbidity and mortality (2, 3), which has resulted in an increase in life expectancy for people living with HIV (PLHIV), from 8–10 years from time of HIV diagnosis (4) to near normal life expectancy compared to the general population (5, 6). As a consequence of ART use, PLHIV have experienced decreases in AIDS-related complications, however, are now at a greater risk of developing age-related co-morbidities, including cardiovascular diseases (CVDs) (7), type 2 diabetes (T2D) (8), neurocognitive disorders (9), and cancers (10). This change is particularly relevant for sub-Saharan Africa, where HIV remains highly prevalent, particularly in Eswatini, the country with the highest prevalence in the world, at 27.2% among those age 15–49 years old (11). With the expansion of HIV treatment in sub-Saharan Africa, PLHIV there are showing marked increases in life expectancy (12). The number of older PLHIV (OPLHIV), defined as those ≥ 50 years of age, is growing worldwide (13) and with the number of OPLHIV increasing in sub Saharan Africa at a more rapid pace compared to higher-income countries (14), which has resulted in increased rates of non-AIDS age-related comorbidities (15). This is particularly challenging for countries that have few programs for persons with NCDs (16).

At the interface between HIV and HIV-associated comorbidities are aging trajectories that may be dysregulated in OPLHIV. Although PLHIV are living longer lives, they are aging at a faster rate, which is a likely outcome of the physiological effects of HIV (17). A significant portion of the ART-treated population display aging-associated phenotypes and morbidities at a higher rate and at a significantly younger age as compared to the general aging population (18), including frailty (19), reduced bone density (20), and age-related diseases (CVDs, T2D, metabolic dysfunction, dementias, etc.) (21–24). Although there is a paucity of data, with the increase in ART treatment access, similar trends have been observed in regions of sub-Saharan Africa (15, 25, 26). These differences in the aging process and the earlier onset of age-related phenotypes among OPLHIV may arise from differences in biological aging, the accumulation of damage to tissues and cells throughout the body as the aging process proceeds across the life-course; a process that is affected by environmental influences (pollutants, chemicals, lifestyle behaviors, infections, *etc.*) (27, 28).

DNA methylation (DNAm), the addition of a methyl group to the 5’ position of cytosine at a cytosine-guanine dinucleotide (CpG), is an epigenetic modification involved in regulating cellular phenotype via gene regulation without changes to the genotype (29). Epigenetic mechanisms, including DNAm, have gained considerable attention in biomedical research given that these epigenetic markers are responsive to environmental influences (*e.g.*, lifestyle factors, infections, chemical exposures, *etc.*) and can regulate underlying cellular functions via transcriptional regulation, which when dysregulated may underlie disease pathogenesis. Importantly, epigenetic clocks were developed to predict chronological aging, such as the first-generation Horvath (30) and Hannum clocks (31) (*i.e.*, epigenetic aging), and the second-generation clock PhenoAge (32) and GrimAge clocks (33), used as biomarkers of biological aging, and, finally, the pace of biological aging clock, DunedinPACE (34). Higher epigenetic age as compared to chronological age, termed epigenetic age acceleration (EAA), has been associated with a wide range of age-related diseases and mortality (35–37). Further, evidence suggests HIV infection may increase epigenetic age by more than 5 years in untreated individuals (38) and despite ART and viral load suppression which have been shown to slow biological aging, it remains relatively increased (39). Although it is generally accepted that biological aging is accelerated in OPLHIV, little is known about biological aging in OPLHIV in sub-Saharan Africa, a region disproportionately affected by HIV.

Additionally, in response to the growing epidemic of non-AIDS comorbidities in OPLHIV, strategies have been implemented that are targeted at mitigating, preventing, and managing age-related diseases in OPLHIV (40, 41). However, in lower-income countries, programs focused on the prevention of comorbidities in PLHIV are limited for several reasons, including lack of resources, including trained staff, integration of programs with health care systems, institutional support (*e.g.*, government, department of health, facilities, *etc.*), and material resources (*e.g.*, educational materials, equipment for HIV care, *etc.*) (42). The limited programs to address NCD in PLHIV likely stem from–in addition to the lack of resources–HIV programs in sub-Saharan Africa that are primarily focused on getting individuals diagnosed, on ART treatment, and virally suppressed (43). Even in uninfected populations, prevention of multimorbidity through interventions is inadequate due to economic constraints (44). By investigating underlying variables associated with HIV and lifestyle and health behaviors that are associated with the aging process, this may inform the shaping of strategies to mitigate HIV-associated comorbidities in lower-income countries.

In this study, we sought to investigate biological aging among OPLHIV on ART in Eswatini by assessing whether HIV-related factors affect the aging process and to determine whether certain modifiable lifestyle and quality of life factors modify this relationship.

## 2. Methods

### 2.1 Study Population

Participants for this study were recruited in an exploratory study of OPLHIV in Eswatini, detailed elsewhere (45). In brief, a convenience sample of 50 PLHIV age ≥ 50 years receiving care at the Mankayane Government Hospital in Manzini region of Eswatini were recruited from October 2016 to January 2017. All participants provided written, informed consent. The study was approved by the Eswatini Directorate of Health Services/Public Health, the Eswatini Ethics Committee, and the Columbia University Irving Medical Center Institutional Review Board.

### 2.2 Data Collection

Quantitative interviews were conducted, and clinical and sociodemographic information were self-reported and abstracted from paper and electronic-based medical records for each participant. Trained staff collected sociodemographic information (*e.g.*, age, education, employment/occupation, financial and medical support, *etc*.), medical history, HIV and sexually transmitted disease information, lifestyle behaviors (*e.g.*, physical activity, smoking, diet), psychosocial behavior (*e.g.*, depressive symptoms), contraceptive use, NCD history, injuries, violence, social support, and quality of life measurements.

To assess quality of life the Short Form Health Survey (SF-36) was used (46), which is a 36-item questionnaire that measures self-reported quality of life by assessing responses across several functional domains, including vitality, physical function, bodily pain, general health perception, physical functioning, emotional functioning, social functioning, and mental health/emotional well-being with each response scored from 0 (low health status) to 100 (high health status). Self-reported dietary intake was measured using the CORE physical activity and diet questions from the WHO STEPwise approach to surveillance (STEPS) survey (47). Average daily dietary intake was calculated based on the number of fruits and/or vegetable servings eaten on a typical day divided by the number of servings eaten in a week; and weekly dietary intake was the total of fruits and/or vegetables, calculated as the number of servings in a typical day multiplied by the number of days fruits/vegetables are eaten in a week. Physical activity was also assessed using the STEPwise approach. Here, we included self-reported physical activity as cumulative physical activity in any given week, which included work-related physical activity (number of days of vigorous- and moderate-intensity work-related activities × number of minutes on any given day of vigorous- and moderate-intensity work-related activities), travel-related physical activity (*i.e.*, number of days per week spent biking or walking to work × number of minutes spent biking or walking to work), and leisure time physical activity (number of days of vigorous- and moderate-intensity leisure physical activity × the number of minutes on any given day of vigorous- and moderate-intensity leisure physical activity). Similarly, self-reported recreational/leisure time physical activity minutes were calculated by the number of days in a week multiplied by the number of minutes of moderate and vigorous physical activity. HIV-related factors included self-reported (age at HIV diagnosis, age at ART initiation) or abstracted from clinical charts (WHO HIV clinical stage at the most recent visit, CD4^+^ cell count (cells/mm^3^) at ART initiation). Finally, the number of years since HIV diagnosis and number of years on ART were determined as the difference between age at HIV diagnosis and age at ART initiation with the age at most recent visit.

### 2.3 DNA methylation Preprocessing, Normalization, and Quantification

Each participant had dried blood spot (DBS) specimen obtained using a finger prick; five separate drops (75–80 microliters each) of blood were collected on a Protein Saver card. DNAm was quantified from the DBS that were collected from 46 of the initially recruited 50 participants following standardized protocols; two participants had low-quality DNA collected, one participant was missing abstracted data, and one participant was missing key self-reported questionnaire data. Blood spots were purified for genomic DNA. DNA was bisulfite-converted and hybridized to Illumina’s Infinium MethylationEPIC BeadChip (850K; Illumina, Inc. San Diego, CA, USA), improving on the coverage, reproducibility, and accuracy from its predecessor the HumanMethylation450 BeadChip, and allowing for the quantification of DNAm at > 850,000 CpGs at single-nucleotide resolution across the genome, covering > 99% RefSeq genes. Raw DNAm IDAT files were generated and loaded into the R statistical environment (version 4.2.2) using the *minfi* package in R (48). Initial quality control removed one sample whose mean detection *p*-value across all probes was unreliable (detection *p*-value ≥ 0.01). Next, normalization was performed using the *ENmix* package in R for background correction using a flexible exponential-normal mixture distribution along with a truncated normal distribution to reflect background noise, followed by a dye-bias correction method using RELIC, which corrects for dye-bias across the array between the two color channels of the 850K (49, 50). Next, we performed inter-array normalization using the quantile normalization for methylation intensities between samples. One sample out of the 46 collected that lacked typical bimodal distribution was also excluded from downstream analyses. Next, we performed probe filtering to remove low quality CpG probes across samples and removed CpG probes when detection *p*-values were not significant against the background (detection *p*-value > 0.01) in one or more individuals, resided on sex chromosomes, harbored known single-nucleotide polymorphisms (SNPs) in the probe sequence, and were cross-reactive. Finally, due to the differential abundance in type I and type II probes on the 850K, we performed probe-type bias adjustment using the Regression on Correlated Probes (RCP) normalization method within the *ENmix* package, which recalibrates type II probes to nearby type I probes. From 45 participants, DNAm values (β-values) were thus generated for 763,250 CpGs, which is based on the ratio of the methylated CpG probe to the total methylation state of the probe (methylated + unmethylated probe) resulting in a DNAm proportion from 0.0 (unmethylated) to 1.0 (fully methylated). Due to potential confounding caused by differences in cell-type heterogeneity that underlies epigenetic variability in heterogenous samples (*e.g.*, blood spots, whole blood, *etc*.) (51), we determined cell-type proportion of each blood sample (CD4^+^ T cell, CD8^+^ T cell, NK cell, monocyte, B cell, neutrophil), we used the Houseman’s projection method employed in the *estimateCellCounts* function in the *minfi* package (52).

### 2.4 Epigenetic Clock Calculation

In order to estimate participants’ epigenetic age, we included two second-generation clocks that are shown to predict age-related phenotypes and lifespan, including PhenoAge (32) and GrimAge (33), both of which differ from the first-generation epigenetic clocks that were trained to predict chronological age, including the pan-tissue Horvath clock (30) and whole blood-based Hannum clock (31). Due to one participant’s epigenetic age deviating by more than 50 years compared to chronological age, we removed this participant from further analyses. Biological aging trajectories were defined as the residuals between regressing chronological age on the estimated epigenetic age. When the resulting residual was positive, participants’ epigenetic age was considered accelerated as compared to their chronological age (*i.e.*, EAA) and negative values represented epigenetic age deceleration. Finally, we employed the DunedinPACE clock, which is intended as a DNAm-based biomarker that measures the pace of aging (34).

### 2.5 Statistical Analyses

Descriptive statistics for participants who had reliable DNAm data available (n = 44) were calculated as median (1st quartile, 3rd quartile) for continuous variables and counts (%) for categorical variables.

For all statistical analyses, we focused on the second-generation PhenoAge and GrimAge clocks, given the association with age-related morbidity and mortality, but have included first-generation clocks as supplementary. To test whether HIV-related variables were associated with EAA in OPLHIV in Eswatini, we first performed linear regression analyses between HIV-relevant variables as the predictor variables [number of years living with HIV (years), age at HIV diagnosis (years), number of years on ART (years), age at ART initiation (years), CD4^+^ cell count at ART initiation (cells/mm^3^), and whether ART was missed at least once in a given month (yes or no)] and biological aging changes as the outcome variable (*i.e.*, residuals of epigenetic age metrics and chronological age), adjusting for relevant variables selected *a priori*, including age (years), sex (male or female), educational attainment (none, primary school, secondary school, high school, tertiary school), and smoking status (never or ever), and immune cell type composition calculated from DNAm data (CD4^+^ cells, CD8^+^ cells, NK cells, B cells, monocytes, and neutrophils).

To investigate whether health and lifestyle factors modified the relationships between HIV-related variables and biological aging, we first utilized linear regression analyses to test the association between health and lifestyle factors (*i.e.*, average daily dietary intake, weekly dietary intake, total physical activity, and SF-36) as the predictor variable and biological aging as the outcome. Next, we employed four separate models to determine whether lifestyle and perceived health factors modified the relationship between HIV-related characteristics and biological aging, including, 1) a model that adjusted for *a priori*-selected covariates (Model 1); 2) Model 1 with further adjustment for average daily dietary intake servings (Model 2); 3) Model 1 with further adjustment for total physical activity (total minutes of moderate and vigorous work, travel/commute, and recreational/leisure activity in a week; Model 3); and, 4) Model 1 with further adjustment for self-reported quality of life based on the SF-36 questionnaire (Model 4). Statistical significance was defined as *p* < 0.05. Linear regression β-estimates (95% Confidence Intervals [CIs]) were calculated in R (version 4.2.2) and graphical figures were created using Prism (version 8; Dotmatics, Boston, MA).

## 3. Results

### 3.1 Participant Characteristics

Descriptive statistics of participants included in this analysis (n = 44) are provided in Table 1. The median age (interquartile range [IQR]) was 59 (54, 66) years, slightly more than half of the participants were female (24 participants [54%]), the average weight was 75 (66, 87) kg, and the highest education attainment for most participants was primary school (grades 1–7; 23 participants [52%]). Participants had a median age of 52 (47, 57) years when diagnosed with HIV and had been living with HIV since diagnosis for a median of 7 (4, 8) years at the time of study enrollment. The median age at ART initiation was 53 (49, 59) years old and participants had been on ART for a median of 6 (3, 8) years. Most of the participants [40 (91%)] were categorized as WHO stage 1 (asymptomatic). Median CD4^+^ cell counts at ART initiation was 223 (98, 308) cells/mm^3^, and all participants had viral load suppression (< 1000 copies/ml) at enrollment in the study. Nine participants (21%) reported missing ART at least once in the last month. The median SF-36 quality of life score was 69 (53, 90). Participants reported consuming 0.8 (0.6, 1.3) fruits and/or vegetable per day, and no participants met the WHO standard vegetable and fruit intake per week. Participants had a median of 855 (390, 2220) minutes of total physical activity per week, with the majority through their work and commuting. Sixteen participants (36%) were past smokers, and three participants are current smokers (7%).

### 3.2 HIV status and biological aging

**Figure 1:**
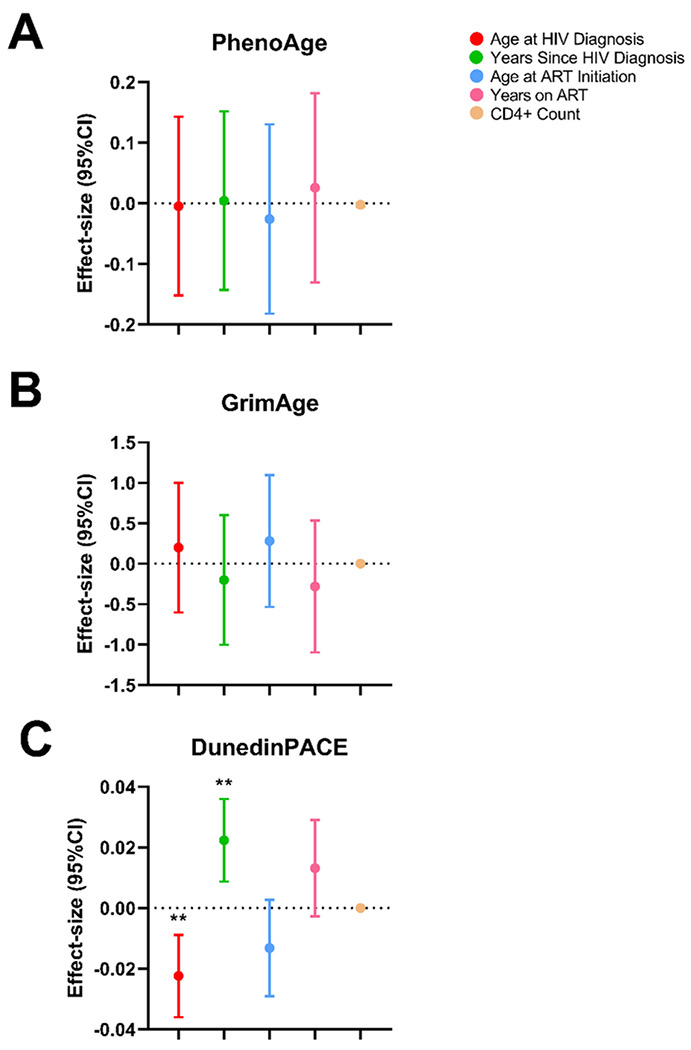
Effect-sizes for association between clinical variables of HIV status and epigenetic clocks from older people living with HIV (OPLHIV) (N=44), Eswatini, 2016-17. Effect estimates (95% Confidence Interval [CI]) for the change in biological calculated from dried blood spots from OPLHIV using HIV status-related variables, including: age at HIV diagnosis (red), years since HIV diagnosis (green), age at antiretroviral therapy (ART) initiation (light blue), years on ART (pink), and CD4+ count at ART initiation (tan). **A.** Depicts change in PhenoAge epigenetic age acceleration (EAA), and **B.** depicts change in GrimAge EAA, and **C.** depicts change in DunedinPACE, pace of biological aging. All plots are adjusted for age, sex, educational attainment, and past smoking status. Significance taken at *p*<0.05, represented by *; *p*<0.05 * and *p*<0.01 **.

Descriptive statistics of epigenetic age calculated from the PhenoAge and GrimeAge clocks, and DunedinPACE are detailed in Table 1. Participants were generally epigenetically older when using the PhenoAge clocks, having a median (IQR) age of 68 (63, 77) years as compared to a chronological age of 59 (54, 66) years, with a difference of 10 years. Participants were epigenetically younger using the GrimAge clock at 56 (50, 61) years as compared to chronological age, with a difference of 3 years compared to chronological age. Given the differences observed between epigenetic age and chronological age, we sought to determine whether HIV-related variables were associated with differences in biological aging. Overall, we found no significant association between HIV-related variables with first-generation clocks (Horvath and Hannum; Supplementary Table 1) nor the second-generation PhenoAge and GrimAge clocks (Fig. 1.A–B; Supplementary Table 1) (all *p* > 0.05). Using the DunedinPACE clock, we observed that the age at which participants were diagnosed with HIV was significantly negatively associated with the pace of aging (−0.02 [−0.04, −0.01], *p* = 0.002; Fig. 1.C; Supplementary Table 1) and the number of years since diagnosis with HIV was significantly positively associated with higher pace of aging (0.02 [0.01, 0.04], *p* = 0.002 Fig. 1.C; Supplementary Table 1).

### 3.3 Health and lifestyle factors and HIV-associated biological aging

**Figure 2:**
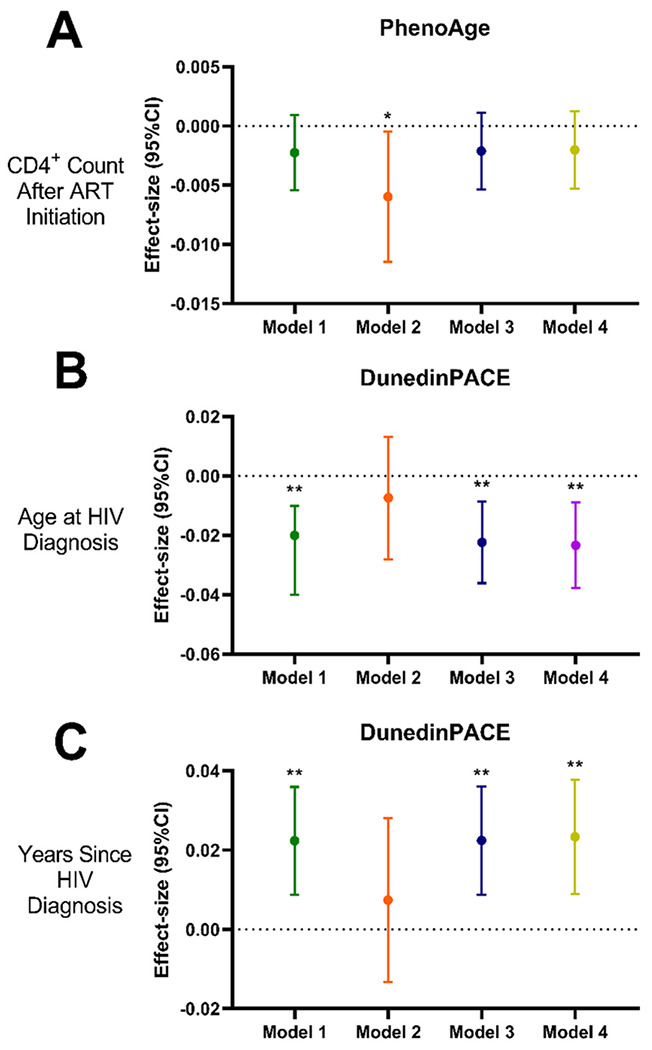
Effect-sizes for the associations between HIV status variables and epigenetic clocks adjusted for lifestyle and quality of life factors. Effect estimates (95% CI) for the change in **A.** PhenoAge residuals by CD4+ Count at ART Initiation, **B.** DunedinPACE by Age at HIV diagnosis, and **C.** DunedinPACE by Years Since HIV Diagnosis. Each plot represents effect estimates for four separate models: Model 1 (Green; covariates: age, sex, education level, past smoking status); Model 2 (Orange; Model 1 + weekly dietary servings of fruits/vegetables); Model 3 (Navy; Model 1 + total physical activity [work related moderate/vigorous + commuting + leisure/recreational moderate/vigorous activity]); Model 4 (Green-Yellow; Model 1 + SF-36 questionnaire [perceived quality of life]). Significance taken at *p*<0.05, represented by *; *p*<0.05 * and *p*<0.01 **.

We next investigated whether quality of life and lifestyle behaviors were associated with biological aging (Supplementary Table 2). We observed a significant positive association between the average daily intake of fruits and vegetables, and DunedinPACE (0.12 [0.03, 0.22]; *p* = 0.01). However, there was no association noted with weekly servings of fruits and vegetables (−0.01 [−0.02, 0.00], *p* = 0.09). There were no significant associations for none of first-generation or second-generation clocks with lifestyle and quality of life characteristics (all p > 0.05) (Supplementary Table 2). Given the association between dietary factors and biological aging in OPLHIV, health and lifestyle behaviors may modify the relationship between HIV status and biological aging trajectories (Fig. 2. A–C; Supplementary Table 1). Using the first-generation clocks, we found no change in biological aging and HIV-related variables in any models (Supplementary Table 1). The finding of significant negative association between age at HIV diagnosis and biological aging using DunedinPACE in Model 1 (−0.02 [−0.04, −0.01], *p* = 0.002; Fig. 1.C & 2.C), persisted after additional adjustment for SF-36 (Model 3: −0.02 [−0.04, −0.01], *p* = 0.003) and total physical activity (Model 4: −0.02 [−0.04, −0.01], *p* = 0.003). However, this relationship was attenuated when adjusting for the average daily intake of fruits and vegetables (Model 2: −0.01 [−0.03, 0.01], *p* = 0.44). Similarly, we found that the number of years since diagnosed with HIV was significantly associated with increased pace of aging using the DunedinPACE clock in all models (Model 1: 0.02 [0.01, 0.04], *p* = 0.002; Model 3: 0.02 [0.01, 0.04], *p* = 0.003; Model 4: 0.02 [0.01, 0.04], *p* = 0.003) except when it was adjusted for average daily dietary intake (Model 2: 0.01 [−0.01, 0.03], *p* = 0.44). Next, we observed CD4^+^ cell counts at time ofwas not significantly associated with changes in PhenoAge EAA (Model 1: −0.02 [−0.05, 0.01], *p* = 0.16; Model 3: −0.02 [−0.04, 0.01], *p* = 0.19; Model 4: −0.02 [−0.04, 0.01], *p* = 0.21) except in models adjusting for average daily dietary intake of fruits/vegetables (Model 2), where CD4^+^ cell counts was negatively associated with PhenoAge EAA (−0.05 [−0.10, 0.00], *p* = 0.04).

## 4. Discussion

Our results show that the age at HIV diagnosis and the duration since HIV diagnosis are associated with differences in the pace of biological aging. HIV diagnosis at older age showed a marked reduction in the pace of biological aging, and conversely, those living with HIV for a longer duration since HIV diagnosis displayed increased pace of biological aging. Additionally, we found that the average daily intake of fruits and vegetables was associated with decrease in the pace of biological aging and modified the relationship between HIV-related variables, PhenoAge EAA, and DunedinPACE. Taken together, these results suggest that HIV-related variables may differentially affect the rate of epigenetic or biological aging in OPLHIV in Eswatini, and lifestyle behaviors may have a protective effect on these trajectories in aging.

Prior to DNAm-based measures of biological aging, several studies have reported increased incidence of age-related phenotypes and biological processes associated with aging in PLHIV, which occurred at a significantly younger age compared to non-infected counterparts (17, 53, 54). This has been further established with the recent improvement in epigenetic clocks, with results showing that epigenetic age is accelerated in PLHIV compared to HIV-uninfected individuals, although most of these studies were conducted in higher-income countries (38, 55). In our study, we also found epigenetic age is accelerated in OPLHIV in Eswatini, which is consistent with previous evidence on biological aging in PLHIV (38, 55). Additionally, our results showed that those who were diagnosed with HIV at an older age–and potentially living with HIV for a shorter duration prior to enrollment in this study–exhibited decreases in the pace of biological aging. HIV infection has extensive effects on immune cellular activity and function, including, most notably, CD4^+^ cell depletion (56), and such changes in immune cell activity are characteristic of biological aging (57). Even with early ART administration, immune cell functions are incompletely restored, and chronic adverse immune-related phenotypes persist (58, 59). Low-grade, chronic inflammation is a significant feature of biological aging, termed by some as ‘inflammaging’, and it can have detrimental effects on physiology and cellular functions, and has been linked to age-related disease pathogenesis (60, 61). Likewise, diagnosis of HIV at an older age may have occurred at a time when there was widespread access to ART in Eswatini enabling rapid initiation of such treatment, compared to those that were diagnosed at a younger age and potentially had lived longer with untreated HIV during years with limited access to ART in the country (62). Notably, biological aging was accelerated in PLHIV that have untreated HIV infection (63) and initiation of ART may decelerate biological aging (64). Taken together, it is possible that the longer duration of living with untreated HIV may elicit long-term effects on biological aging trajectories through inducing chronic proinflammatory phenotypes, while timely initiation of ART may benefit aging trajectories by attenuating the effects of untreated HIV.

Despite the use of ART, the risk of developing comorbidities in OPLHIV remains relatively high, which is likely due to one or more factors (*e.g.*, effects of HIV, ART, lifestyle behaviors, etc.) that influence the aging process. To address this, several interventions have been implemented to reduce HIV-associated comorbidities. For example, the PRECluDE Consortium, funded by the National Institute of Health, has modeled several interventions and clinical trials for PLHIV for CVD prevention (40) and the REPRIEVE phase 3 trial has shown marked reductions in CVD risk using pitavastatin calcium in PLHIV (65), although these efforts have yet these have yet to be implemented in sub-Saharan Africa. Mounting evidence suggests that health-promoting behaviors are linked to slower aging (66, 67). Thus, developing practical strategies to mitigate comorbidities in OPLHIV will be valuable in lower-income countries where resources are constrained. Indeed, practical interventions have been developed for prevention and management of chronic diseases in OPLHIV, including physical activity and nutritional interventions (68, 69). While we found no association nor modification by physical activity and quality of life (SF-36) with biological aging, we did observe that dietary intake of fruits and vegetables was associated with the pace of biological aging and may have a modifying role in the association between HIV-related factors and biological aging trajectories. The lack of effect of physical activity may be due to the fact that most participants accumulated their physical activity from work-related activities, whereas previous evidence suggested that leisure/recreational may have beneficial effects on biological aging and overall health as opposed to the opposite effect with work-related activity (70, 71).

In our study, we found that dietary intake of fruits and vegetables modified the relationships between age at HIV diagnosis, years living with HIV since diagnosis, and CD4^+^ cell count. To our knowledge, there is no data on the association of dietary interventions and biological aging trajectories in PLHIV. However, previous evidence in HIV-uninfected individuals suggests that dietary interventions can have beneficial effects on biological aging, decelerating, and potentially reversing, biological aging, including interventions focused on higher quality foods and specific types of diets (*e.g.*, Mediterranean Diet, DASH Diet, *etc*.) (72–76). For associations between age at HIV diagnosis and years since HIV diagnosis, we found contradictory results when including dietary intake in our models, although both reduced the relative change in biological aging. This may suggest that the higher intake of fruits and vegetables can have a beneficial effect on aging in PLHIV, particularly those with longer time since HIV diagnosis, by counteracting the adverse long-term effects of HIV and subsequent toxicity caused by long-term adherence to ARTs on the epigenome with the beneficial effects of healthy dietary behaviors (77–80). Conversely, when someone becomes HIV infected later in life, dietary patterns may be less effective in affecting aging trajectories, which may be due to elderly individuals having different nutritional needs, metabolic changes, and physiological changes that effect digestion (81). The significant negative association between CD4^+^ cell counts and lower PhenoAge EAA when adjusting for dietary patterns, which was not observed in any other model, suggests a protective role for diet on immune function, which could precede aging and age-related disease risk. Evidence suggests nutrient quality both predicts CD4^+^ cell counts and is associated with a lower risk of mortality in PLHIV (82), and diet quality is associated with lower CD4^+^ cell count (83). This suggests that diet may not only improve immune cell function, but that this rebound of CD4 + cell count may be reflected in improved biological aging trajectories. Altogether, our findings suggest a beneficial role for diet on the relationship between HIV-related variables and biological aging trajectories and supports the utilization of practical dietary interventions that would be beneficial for modifying aging trajectories and NCD risk for PLHIV in resource-constrained regions of the world.

While this study is, to our knowledge, the first to investigate biological aging in OPLHIV in Eswatini, it has several limitations. First, this is an exploratory study and thus the findings are constrained by the limited sample of participants (45). As our study used a cross-sectional design, we could not ascertain whether biological aging trajectories changed over time, especially from early stages of infection (untreated) to ART initiation and viral load suppression. Likewise, these data were limited to the HIV-related factors included in the study. For example, time since HIV diagnosis is not reflective of time since HIV infection. Accumulating evidence suggests that HIV-related factors prior to ART initiation may be important to biological aging trajectories and long-term health outcomes (63, 84). Future research should examine changes in biological aging trajectories longitudinally, prior to ART initiation. Finally, our study was performed exclusively in OPLHIV in Eswatini, thus, the findings may not be generalizable to other populations or regions in sub-Saharan Africa. Although our study had these limitations, this exploratory study showed compelling evidence that may underlie aging trajectories in OPLHIV in a lower-income country which may provide a foundation for future studies to further explore the molecular mechanisms of aging in PLHIV in sub-Saharan Africa and other resource limited settings.

## 5. Conclusions

We found evidence that HIV-related variables are associated with biological aging in OPLHIV on stable ART in Eswatini, a country with limited resources in sub-Saharan Africa. We also showed that a modifiable risk factor, diet, may be important in influencing aging trajectories in OPLHIV, which may unveil potential avenues for practical interventions that may be relevant to slowing aging trajectories and NCD risk in OPLHIV in regions with limited resources. With increased access to ART in Eswatini, and other regions of sub-Saharan Africa, life expectancy of PLHIV will continue to increase with an expanding older population living with HIV, with increasing risk of NCD. Developing practical interventions that are aligned with “healthy aging” will be critically important.

## Figures and Tables

**Table 1:** Participant characteristics from pilot study of OPLHIV in Eswatini included in DNA methylation analyses

Variable	Participants (n = 44)
Age (years) [median (IQR)]	59 (54, 66)
Sex	
Female	24 (54%)
Male	20 (46%)
Weight, at recent visit (kg)	75 (66, 87)
Education	
None	5 (11%)
Primary School Grade 1–7	23 (52%)
Secondary School Form 1–3	12 (27%)
High School Form 4–5	4 (9%)
Years Since HIV Diagnosis (years)	7 (4, 8)
Age at HIV Diagnosis (years)	52 (47, 57)
ART Duration (years)	6 (3, 8)
Age at ART Initiation (years)	53 (49, 59)
WHO Clinical Stage of HIV, at recent visit (Stage 1)	40 (91%)
CD4 + T Cell Count at ART Initiation (cells/uL)	223 (98, 308)
Number of Times Missed ART, month (≥ 1 day)	9 (21%)
SF-36 Health Questionnaire (0-100 Scale)	69 (53, 90)
Average Fruits and/or Vegetables per day (servings)	0.8 (0.6, 1.3)
Dietary Intake of Fruits and/or Vegetables, weekly (servings)	10 (7, 14)
Eat Processed Food High in Salt, always/often	19 (43%)
Physical Activity	
Total Physical Activity, week (minutes)	855 (390, 2220)
Recreational/Leisure Time Physical Activity (≥ 30 minutes, week)	2 (5%)
Smoked Tobacco Ever (count [%])	16 (36%)
*Immune Cell Composition (% of Total Composition)*	
CD4 + T	7% (5, 10)
CD8 + T	23% (18, 27)
Natural Killer Cell	6% (5, 9)
B Cell	8% (5, 9)
Monocyte	10% (9, 12)
Neutrophil	51% (44, 55)
*Epigenetic Aging Metrics*	
PhenoAge Epigenetic Age (years)	68 (63, 77)
GrimAge Epigenetic Age (years)	56 (50, 61)
DunedinPACE (pace to ref. of 1.0)	1.2 (1.1, 1.3)

Continuous variables: mean (1st quartile, 3rd quartile); categorical variables: count (%)

WHO clinical stage of HIV: stage 1 (asymptomatic) to stage 4 (AIDS)

SF-36 Health Questionnaire: 36-item health survey to assess overall quality of life; 0 (low) to 100 (high)

WHO recommends 35 servings of fruits and/or vegetables per week

Total physical activity calculated as the number of minutes of work-related (moderate, vigorous), commuting-related (bike, walk), and recreational/leisure time (moderate, vigorous) physical activity

Immune cell composition calculated using the Houseman’s projection method for DNA methylation data

Abbrv.: ART, antiretroviral therapy; IQR, interquartile range; WHO, World Health Organization

## Data Availability

The data that supports findings from this study are available upon reasonable request from TGH. The data is part of an on-going collaboration between ICAP and the Eswatini Ministry of Health, and thus, is not currently publicly available.
